# Twenty years after: the beautiful hypothesis and the ugly facts

**DOI:** 10.1186/s40880-016-0087-1

**Published:** 2016-02-24

**Authors:** Francesco Pezzella, Kevin Gatter, Chao-Nan Qian

**Affiliations:** Nuffield Division of Clinical Laboratory Science, Radcliffe Department of Medicine, John Radcliffe Hospital, University of Oxford, Oxford, OX3 9DU UK; State Key Laboratory of Oncology in South China, Collaborative Innovation Center for Cancer Medicine, Sun Yat-sen University Cancer Center, Guangzhou, 510060 Guangdong People’s Republic of China

**Keywords:** Angiogenesis, Vasculogenic mimicry, Vessel co-option, Treatment resistance, Targeted therapy

## Abstract

The limited clinical benefits from current antiangiogenic therapy for cancer patients have triggered some critical thoughts and insightful investigations aiming to further elucidate the relationship between vessels and cancer. Tumors need blood perfusion but there are mounting evidences that angiogenesis alone does not explain it in all the neoplasms. In this editorial, for a special issue on tumor and vessels published in the *Chinese Journal of Cancer*, we briefly introduce the history of the evidences that solid tumors can sometimes obtain blood perfusion by alternative approaches other than sprouting angiogenesis, i.e., vessel co-option and vasculogenic mimicry. This editorial provides also the links to several most recently published discoveries and hypotheses on tumor interaction with blood vessels.

“The great tragedy of science–the slaying of a beautiful hypothesis by an ugly fact.” Thomas Huxley.

Heraclitus of Ephesus, one of the most distinguished Greek thinkers, had at the core of his philosophy the idea that everything changes. According to Plato, “Heraclitus, I believe, says that all things go and nothing stays, and comparing existents to the flow of a river, he says you could not step twice into the same river” (*Cratylus* 402a = DK22A6). Twenty years after the special issue on angiogenesis published in the *European Journal of Cancer* [1], we are stepping into another special issue, concerning the relationship between cancer and blood vessels, of the *Chinese Journal of Cancer* and things have certainly changed! Perhaps the first thing to be noticed is that this time we will not get a single bounded issue with all the contributions inside. We will rather end up with a website link leading to the collection of papers, to be posted throughout the next 12 months or so, on a server. The journal has changed as well, from the *European Journal of Cancer* to the *Chinese Journal of Cancer*, as a result of an ever-increasing wide but tightly connected research community. However, what about the subject of the two issues? In 1996, it was firmly about the fundamental role of angiogenesis, i.e., the formation of new vessels, in cancer but now is going to be about cancer and blood vessels—not only newly formed but also pre-existing, plus vascular-mimicking structures.

The fundamental question behind all this is how the cancer cells get provided with the necessary oxygen and nutrients in order to stay alive and growth. The prevalent theory at the time of the *European Journal of Cancer* special issue was the one, introduced in 1971 by Folkman [2], that tumor growth is strictly angiogenesis-dependent. However, the discovery that tumor can also grow without angiogenesis, by co-opting pre-existing vessels in humans [3–7] and in mice [8] plus the possibility, known as “vascular mimicry,” for the very same neoplastic cells to form vessel-like structures able to provide blood perfusion, has demonstrated that this is not always the case [9]. These observations provide a new aspect of the interaction between vessels and tumors, shed new light on the biology of the latter, and have implications for resistance to antiangiogenic drugs and development of new targeting strategies. The relationship between cancer and blood vessels is emerging as much more complex than until now thought; while angiogenesis remains a fundamental part of it, it is clearly not the all story.

But how new are the findings that cancer can also grow exploiting pre-existing vessels in complete absence of angiogenesis? It would be surprising that such important role played by the normal vessels of the body in supporting the spread of cancers had been so far ignored. As a matter of fact it was not ignored as the idea that cancer must have had a tight relationship with vasculature is an old one and different theories have been discussed throughout the time. Kolin et al. [10] described primary lung carcinomas “often growing mainly in air spaces and preserving the pulmonary framework as their stroma.” In the General Pathology textbook edited in 1962 by Florey [11], Ritchie wrote, in the chapter on “The classification, morphology, and behaviour of the tumors,” that “One of the principal functions of the stroma is to provide a blood supply within the tumor mass.” but “Sometime a tumor will supplement or replace the stroma by using pre-existing structures. For example occasional tumors in the lung grow round the alveoli using the alveolar walls in place of stroma.”

Reading backwards, in his 1934 book “The spread of tumors in human body” Willis [12] wrote that “Intra alveolar growth of tumors in the lung is a characteristic and frequent mode of extension” in which “the plugs of growth occupying the air sacs are themselves avascular, the septal walls constituting the only stroma of the tumors.” Willis credited for the original description of these tumors an article published in 1861. In that study entitled “Zwei falle von carcinosis acuta miliaris” (Two cases of acute miliary carcinomatosis), Erichsen [13] described how the neoplastic cells in lung tumors occupy the alveolar spaces but no new vessels can be seen; he also illustrated his point with a remarkable “Camera Lucida” drawing. The diverse findings from different investigators, which try to unravel how cancer and vessels interact, have been until now looked at as somehow in contrast among them, but we have nowadays accepted that they are all in the thick of it!

The most immediate consequence of the recognition that malignant cells can exploit pre-existing vessels has been the opening of two new big fields in cancer biology.

The first is, of course, the biology of the non-angiogenic cancer cells: why some of them induce angiogenesis while others can go all the way of exploiting pre-existing vessels and how the biology of these two types of tumors differs. It is worthy noting that in many tumors both angiogenic and non-angiogenic areas are identified [3];

The second is the biology of vessel co-option: how cancer cells do it? Plus the obvious subsequent question: how does this affect treatment with the present day drugs, and can new targets be discovered?

These will be the first papers among those of our “virtual” special issue papers of the *Chinese Journal of Cancer*. Dr. Qian and colleagues [14] discussed vessel remodeling accompanying vessel co-option as well as the different features between vessel-like structure and tubule-like structure. Dr. Paku and his team [15] reviewed the biological features of the mechanisms underlying vessel co-option and other alternative angiogenic approaches in the primary and metastatic tumor lesions. Dr. Pezzella and his team [16] showed that hypoxia occurs in a comparable way in angiogenic and non-angiogenic tumors and proposed a hypothesis that in some, but not in all, cases, initial tissue remodeling and/or inflammation could possibly be one of the second steps, following the establishment of hypoxia, necessary to trigger tumor angiogenesis. In the non-angiogenic tumors, where neo-vascularization fails to occur, hypoxia-inducible factor (HIF) pathway activation could be the driving force toward metabolic reprogramming. Dr. Luo and his group [17] reported that mono-PEGylated endostatin had a promising property on inhibiting tumor angiogenesis. Dr. Cao [18] discussed the possible mechanisms of various antiangiogenic drugs and future development of optimized treatment regimens.

Happy reading!
